# Type 2 diabetes mellitus and the risk of male infertility: a Mendelian randomization study

**DOI:** 10.3389/fendo.2023.1279058

**Published:** 2023-12-12

**Authors:** Xiao-Bin Zhu, Zhi-Hong Niu, Wei-Min Fan, Chang-Sheng Sheng, Qian Chen

**Affiliations:** ^1^ Department of Gynecology and Obstetrics, Reproductive Medical Center, Shanghai Ruijin Hospital, Shanghai Jiao Tong University School of Medicine, Shanghai, China; ^2^ Department of Cardiovascular Medicine, Center for Epidemiological Studies and Clinical Trials and Center for Vascular Evaluation, Shanghai Key Lab of Hypertension, Shanghai Institute of Hypertension, Ruijin Hospital, Shanghai Jiaotong University School of Medicine, Shanghai, China

**Keywords:** type 2 diabetes mellitus, male infertility, erectile dysfunction, Mendelian randomization (MR) analysis, expression quantitative trait loci (eQTL)

## Abstract

**Objective:**

To assess the causal effect of type 2 diabetes mellitus (T2DM) on male infertility (MI) and erectile dysfunction (ED) by Mendelian randomization (MR) analysis.

**Methods:**

Data for T2DM, MI, and ED were obtained from genome-wide association studies (GWAS) involving 298, 957, 73, 479, and 223, 805 Europeans, respectively. We performed univariate MR analysis using MR Egger, Weighted median (WM) and Inverse variance weighted (IVW) methods to assess causal effects among the three. Through the Genotype Tissue Expression (GTEx) database, single-nucleotide polymorphisms (SNPs) that affect the expression levels of T2DM-related genes were located using expression quantitative trait loci (eQTL).

**Results:**

MR analysis showed a significant causal relationship between T2DM and ED (WM, OR: 1.180, 95%CI: 1.010-1.378, *P* = 0.037; IVW, OR: 1.190, 95%CI: 1.084-1.300, *P* < 0.001). There is also a significant causal relationship between T2DM and MI (MR Egger, OR: 0.549, 95%CI: 0.317-0.952, *P* = 0.037; WM, OR: 0.593, 95%CI: 0.400, *P* = 0.010; IVW, OR: 0.767, 95%CI: 0.600-0.980, *P* = 0.034). ED may not cause MI (*P* > 0.05). We also found that rs6585827 corresponding to the PLEKHA1 gene associated with T2DM is an eQTL variant affecting the expression of this gene.

**Conclusion:**

T2DM has a direct causal effect on ED and MI. The level of PLEKHA1 expression suppressed by rs6585827 is potentially associated with a lower risk of T2DM.

## Introduction

Infertility has become a common global problem, affecting 10-15% of couples, with approximately 40% of cases being caused by the male factor (1). Common causes and risk factors of male infertility (MI) have been hypothesized and confirmed in various studies, including erectile dysfunction (ED) (2), smoking, alcohol consumption, and hormonal disorders, etc. (3). An insightful review suggests that MI symptoms may serve as future markers of mortality and health status (4).

Type 2 diabetes mellitus (T2DM) is a heterogeneous disease caused by the interaction of genetic, environmental and other factors. It is the main type of diagnosed DM cases (5, 6), accounting for about 90%-95% (7). In recent years, the incidence of T2DM has been increasing worldwide (8), and T2DM and its complications including ED have become a huge burden on global public health (9). T2DM usually affects male reproductive function at multiple levels, including structural changes in reproductive organs, ejaculation, and ED (10, 11). Potential related factors: diabetes can lead to nerve damage, and nerve damage in sexual organs can lead to ED (12); Diabetes can cause vascular damage, including obstruction and sclerosis, which may affect the ability of blood flow to the penis (13); In addition, diabetes is usually accompanied by chronic inflammation, which may affect various systems of the body, including the reproductive system (14). However, it needs more direct and accurate data to prove that diabetes has an impact on male infertility and ED. It is particularly important to clarify the relationship between T2DM, MI and ED, and to explore the pathogenesis of T2DM.

In recent years, significant progress has been made in identifying genomic regions associated with complex traits and diseases through GWAS. However, a challenge in interpreting GWAS findings is that most of the associated SNPs are located in intergenic regions. It is therefore difficult to infer functional genes and variants in these regions. Expression quantitative trait loci (eQTL) analysis can locate SNPs that affect the expression level of one or more genes, providing an effective and feasible strategy for evaluating the biological mechanism of SNPs in non-coding regions (15). Researchers are using genotype tissue expression (GTEx) data to enhance the functional interpretation of GWAS findings and identification of disease-associated genes to assess tissue-specific gene expression and regulation in many different tissues (16). Therefore, this study conducted Mendelian randomization (MR) analysis through genetic variants related to “type-2 diabetes mellitus”, “erectile dysfunction” and “male infertility” derived from the GWAS database.

Randomized controlled trials (RCTs) should be an ideal study design to confirm the causal relationship between diabetes and ED. However, conducting RCTs in reality faces difficulties. MR minimizes the effects of measurement error and directional causality. Since these instrumental variables (IVs) remain constant after conception and are expected to be free from potential founders, the MR approach overcomes some limitations of traditional epidemiological studies. The MR-Egger approach provides a progressively consistent measure of causal effect, adjusting for horizontal pleiotropy by pooling individual SNP-specific Wald ratios via adaptive Egger regression. The WM (weighted median) method produces progressively consistent causal effect estimates by using the weighted median of the Wald ratios, provided that at least 50% of the variants meet the effective IV of the exclusion limits. The IVW (inverse variance weighted) method is the most widely used and accepted MR method because it is the most effective method in the presence of effective IVs and can also consider heterogeneity in the analysis of causality. Therefore, this study mainly used MR-Egger, WM and IVW, a total of three algorithms for univariate MR analysis to evaluate the causal relationship between T2DM, ED and MI.

## Methods

### Data sources

The data on T2DM, MI, and ED were obtained from three different Genome-Wide Association Studies (GWAS) conducted on European populations, including 298,957 (17), 73,479, and 223,805 (18) individuals, respectively (**Supplementary Table S1**). All summary GWAS data were sourced from the IEU GWAS database (https://gwas.mrcieu.ac.uk/). The GSE9006 dataset analyzed gene expressions in peripheral blood mononuclear cells (PBMCs) from 24 healthy volunteers, 43 individuals with Type 1 Diabetes (T1D), and 12 individuals from the USA population diagnosed with T2D. Additionally, it includes transcriptional level data from 36 individuals from the USA population with T2DM (19) (**Supplementary Table S2**).

### Study design

This project proposes three main hypotheses: 1) SNPs used as IVs are significantly associated with T2DM, ED, and MI phenotypes and reach genome-wide significance thresholds; 2) SNPs are independent of confounding factors; 3) SNPs were only associated with MI and ED through T2DM or ED, but not through other pathways. In addition, expression quantitative trait loci (eQTL) analysis can locate SNPs that affect the expression level of one or more genes, we use the GTEx database (https://www.gtexportal.org/home/) to assess the tissue in many different tissues specific gene expression and regulation.

### Mendelian randomization analysis

First, we selected instrumental variables IVs. Use the clustering threshold (r ^2^ < 0.001, kb = 10000) in the PLINK clustering method to remove SNPs that are biased by linkage disequilibrium (LD), and according to the allele frequency and allele incompatibility of the palindrome (incompatible alleles) low-quality SNPs were removed, and SNPs significantly associated with T2DM, ED and MI phenotypes were retained (*P* < 5×10^-8^). MR analysis was performed with T2DM and ED as exposure factors, and MI and ED as outcome. Additionally, Cochran’s Q statistical tests were performed to confirm heterogeneity among the selected IVs, where less heterogeneity indicates more reliable MR estimates, and horizontal pleiotropy was performed on the MR Egger algorithm test. Leave-one-out sensitivity analyzes can be used to assess the impact of individual SNPs on causality estimates.

### T2DM models

All animals were approved by the Ethics Committee (Institutional Review Board) of Shanghai Ruijin Hospital. To induce T2DM, diabetes was induced by a single intraperitoneal injection of 60 mg/kg streptozotocin (STZ) (Sigma Aldrich, Shanghai; mixed in a freshly prepared cold 0.1 mol/L citric acid cradle, pH 4.2-4.5). Three male SPF grade SD rats (6-7-week-old), body weight (220 ± 20 g), feed them adaptively for one week, fast for 16 h, and inject STZ (60 mg/kg) intraperitoneally. Three uninjected STZ rats were used as the normal control group, and the rats in the control group were injected with citrate buffer intraperitoneally. Three days after STZ infusion, blood glucose levels in tail vein blood were measured using the OneTouch Ultra system (Johnson & Johnson Medical, Shanghai, China), and checked once a week. Only when the blood glucose concentration remains consistently above 16.7 mmol/L and exhibits symptoms such as excessive thirst, excessive eating, frequent urination, and weight loss, can the modeling be considered successful (**Supplementary Figure S1A**) (20). Besides, severe steatosis was observed in the liver tissues of T2DM mice; the hepatic lobule structure was not clear, the volume of liver cells was significantly increased and disordered, and fat vacuoles of different sizes were present in the cytoplasm; intestinal villi were irregular in shape and disorderly in surface arrangement, and some villi were thicker, wider, unequal in thickness; the shape of pancreatic islets in T2DM rats was irregular, the islets were atrophic, the color was pale and gray, and the tissue was thin (**Supplementary Figure S1B**).

### Western blot

The rat semen, penis tissue, and peripheral blood samples frozen in groups in liquid nitrogen were taken out, and the total protein of the samples was extracted with RIPA lysis buffer according to the instructions of the protein extraction kit. The protein solution extracted above was subjected to polyacrylamide gel electrophoresis (SDS-PAGE, 10% separating gel), and then transferred to PVDF membrane, blocked for 30 min at 37°C, and the primary antibody (PLEKHA1 antibody: 10238-1-AP), incubate overnight at 4°C, and after elution of the primary antibody, incubate with the secondary antibody at 37°C for 90 min, use the Odyssey two-color infrared laser imaging system and Alpha software to scan and semi-quantitatively analyze the protein bands, and use mouse anti-β-actin (1:1000) as an internal reference, the relative expression of PLEKHA1 protein was calculated.

### Statistical analysis

MR is based on the principle of random distribution of genetic genes. When the frequency of SNPs is highly consistent with the changes in exposure variables, it can be preliminarily considered that SNPs are related to exposure variables. All statistical tests were two-sided and considered to show statistical significance at *p*-values <0.05. The causal relationship among T2DM, MI and ED was assessed using 3 methods with IVW as the main analysis method. We explored horizontal pleiotropy by the MR-Egger method. Leave-one-out sensitivity analysis and heterogeneity analysis were used to demonstrate the reliability of pleiotropic effects of IVs and to correct abnormal results caused by outliers.

## Results

### Selected SNPs and IVs validation

In this study, the identification and information of genetic variation related to “type-2 diabetes mellitus”, “erectile dysfunction” and “male infertility” comes from the GWAS database. For the GWAS data that are significantly associated with the above disease phenotypes, after excluding LD for SNPs that caused bias and low quality, 58 and 105 SNPs were retained as IVs (*P* < 5×10^-8^), respectively. The genes to which each SNP belonged were further retrieved, and their details were summarized in a table (**Table 1**).

**Table 1:** Details of 58 SNPs.

SNPs	se	P val	β	effect_allele	other_allele	Gene names
rs10244051	0.0062	7.865E-18	0.0479	G	T	NA
rs1065461	0.0074	1.756E-09	0.0388	C	T	TCF19
rs10758593	0.0062	2.5568E-11	0.0393	A	G	GLIS3
rs10842994	0.0081	7.5998E-13	-0.0575	T	C	NA
rs10906115	0.0063	4.958E-08	-0.0339	G	A	NA
rs10965250	0.0093	3.7128E-46	-0.1298	A	G	NA
rs11063069	0.0078	3.435E-10	0.0469	G	A	CCND2-AS1
rs11603334	0.009	1.7231E-24	-0.0881	A	G	ARAP1
rs11708067	0.0078	9.324E-31	-0.0839	G	A	ADCY5
rs12571751	0.0062	3.1383E-22	-0.0559	G	A	ZMIZ1
rs1260326	0.0065	3.1849E-18	0.0543	C	T	GCKR
rs13133548	0.0066	4.9951E-08	0.0334	A	G	FAM13A
rs13266634	0.0068	1.3011E-47	-0.0897	T	C	SLC30A8
rs13389219	0.007	1.321E-29	-0.0715	T	C	COBLL1
rs1359790	0.0073	1.3431E-15	-0.0585	A	G	NA
rs16826069	0.0079	6.97E-09	0.0465	G	A	MACF1
rs1727307	0.0067	6.189E-10	0.0384	G	A	PITPNM2,ENSG00000280381
rs1801212	0.0073	7.0909E-21	0.063	A	G	WFS1,ENSG00000286176
rs2237895	0.0066	1.8289E-38	0.0797	C	A	KCNQ1
rs2395163	0.0078	1.721E-09	0.0488	C	T	NA
rs243021	0.0065	4.5952E-11	0.0402	A	G	MIR4432HG
rs2796441	0.0063	4.8607E-11	-0.04	A	G	TLE1-DT
rs2943641	0.0069	1.4279E-18	0.0603	C	T	NA
rs340874	0.0063	1.4051E-15	0.0486	C	T	PROX1,PROX1-AS1,ENSG00000274895
rs35658696	0.0175	7.4422E-15	0.1222	G	A	PAM
rs35720761	0.0104	8.3119E-16	-0.0721	T	C	THADA
rs4077129	0.0079	3.523E-08	-0.0395	C	T	PIM3
rs4402960	0.0066	8.3138E-49	0.0919	T	G	IGF2BP2
rs4457053	0.0075	3.6788E-13	-0.0466	A	G	ZBED3-AS1,ENSG00000284762,ENSG00000285000
rs4502156	0.0065	9.5521E-11	-0.04	C	T	NA
rs459193	0.0072	6.7066E-15	0.0537	G	A	NA
rs4607103	0.0069	6.759E-10	-0.0415	T	C	ADAMTS9-AS2
rs4812831	0.01	1.321E-08	0.0587	A	G	HNF4A,HNF4A-AS1
rs4886707	0.007	4.321E-09	-0.0388	T	C	NA
rs5015480	0.0065	1.3599E-30	-0.0709	T	C	NA
rs516946	0.0076	9.324E-20	0.0652	C	T	ANK1,ENSG00000253389
rs5219	0.0065	1.523E-22	-0.063	C	T	KCNJ11
rs55834942	0.0085	1.348E-12	-0.0514	A	G	HNF1A
rs58542926	0.0116	3.3558E-15	0.0836	T	C	TM6SF2,ENSG00000267629
rs60980157	0.0079	6.6344E-16	-0.0615	T	C	GPSM1
rs6585827	0.0065	9.531E-09	-0.0361	A	G	PLEKHA1
rs6813195	0.007	1.5329E-12	-0.0461	T	C	NA
rs6905288	0.0065	5.773E-11	0.0401	A	G	NA
rs7177055	0.0072	5.652E-14	0.0528	A	G	NA
rs7202877	0.0099	3.1427E-14	-0.0726	G	T	NA
rs72928978	0.0104	1.838E-08	-0.0479	A	G	TPCN2
rs730497	0.0084	3.069E-08	0.0413	A	G	GCK
rs731839	0.0067	7.4216E-13	-0.0443	A	G	PEPD
rs7501939	0.0064	2.3818E-24	-0.0622	C	T	HNF1B
rs7756992	0.0067	1.3259E-61	0.1073	G	A	CDKAL1
rs7903146	0.0069	1E-200	0.241	T	C	TCF7L2
rs7961581	0.0069	7.246E-09	-0.0386	T	C	TSPAN8
rs8042680	0.0068	1.664E-10	0.0427	A	C	PRC1,PRC1-AS1,ENSG00000284946
rs8108269	0.0072	5.1535E-16	0.0543	G	T	NA
rs864745	0.0065	2.8728E-30	-0.0701	C	T	JAZF1
rs9379084	0.0129	1.4689E-17	-0.0946	A	G	RREB1
rs9388489	0.0067	1.187E-08	0.0345	G	A	ENSG00000286215
rs972283	0.007	1.509E-12	0.0473	G	A	NA

SE, standard error; NA, Not Available.

### The causal relationship between type-2 diabetes mellitus and erectile dysfunction

**Figure 1:**
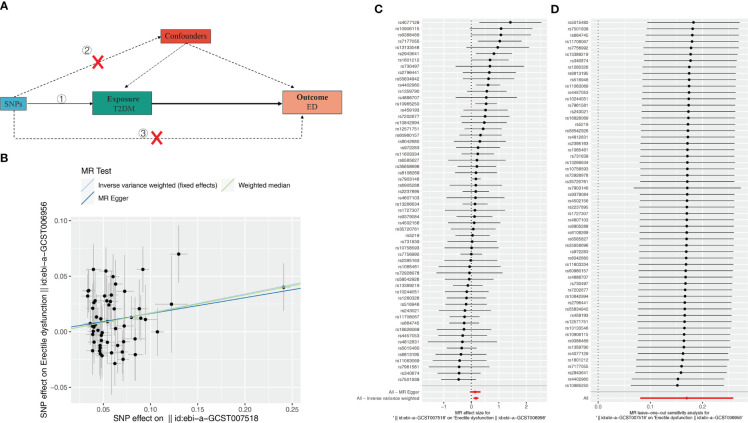
Effect of T2DM on erectile dysfunction (ED). **(A)** Schematic diagram of the steps of the two-sample MR analysis. **(B)** Scatterplot showing the distribution of individual rate estimates for T2DM as a result of ED. Each scatterplot also contains trendlines derived from 3 different MR methods to indicate causality. **(C)** MR analysis forest plot of the association between T2DM and ED. The circles next to each SNP represent causal estimates for each IV, respectively, and the lowest two circles show multiple-instrument MR analysis using Egger regression and inverse-variance weighted methods. Horizontal lines denote 95% CIs. **(D)** MR leave-one-out sensitivity analysis, used to estimate the causal effect of T2DM on ED, each black point represents an IVW, the red point represents the estimated value using all IVs, and the horizontal line represents the 95% confidence interval.

To assess the causal effect between T2DM and ED, we employed three-step two-sample MR analysis in this work, and the results are listed in **Table 2** and **Figures 1A, B**. Our results found a significant causal relationship between T2DM and ED in the European population (WM, OR: 1.180, 95%CI: 1.010-1.378, *P* = 0.037; IVW, OR: 1.190, 95%CI: 1.084-1.300, *P* < 0.001) (**Table 2**, **Figure 1C**).

**Table 2:** Significant causal relationship between T2DM and erectile dysfunction (ED).

exposure	outcome	method	nsnp	β	se	pval	or	or_lci95	or_uci95	Q	Q_pval	egger_intercept	pval_intercept
T2DM	ED	MR Egger	58	0.143	0.098	0.148	1.154	0.953	1.397	63.092	0.240	0.002	0.758
T2DM	ED	WM	58	0.165	0.079	0.037	1.180	1.010	1.379				
T2DM	ED	IVW	58	0.170	0.045	0.000	1.185	1.084	1.295	63.200	0.267		

To evaluate the MR hypothesis in the work, we selected SNPs with a genome-wide significance level of *P* < 5 × 10^-8^ to meet our first condition. Leave-one-out sensitivity analysis showed that deleting of any SNP did not significantly change the results, indicating the reliability of the results (**Figure 1C**). Cochran’s Q test was applied to assess the heterogeneity among the selected SNPs, and the results showed that neither MR Egger nor IVW analysis had statistically significant heterogeneity. No evidence of directional pleiotropy was found as measured by MR-Egger regression (*P* for intercept > 0.176). The above results verified our hypothesis that the SNPs used as IVs were significantly associated with ED, and the causal estimate between T2DM and the risk of ED didn’t receive confounding factors.

### The causal relationship between type-2 diabetes mellitus and MI

**Figure 2:**
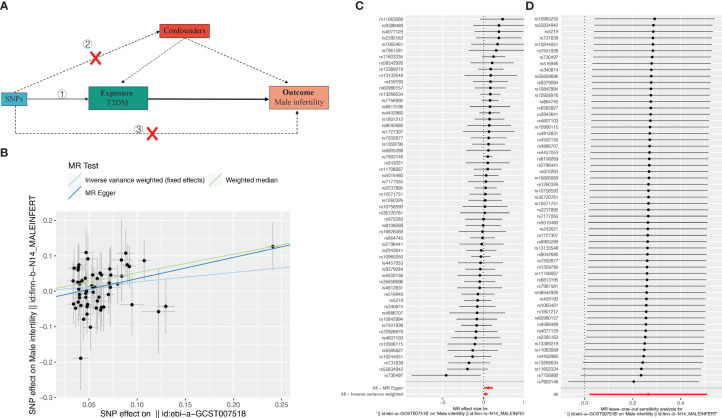
Effect of T2DM on male infertility (MI). **(A)** Schematic diagram of the steps of the two-sample MR analysis. **(B)** Scatterplot showing the distribution of individual rate estimates for T2DM as a result of MI. Each scatterplot also contains trendlines derived from 3 different MR methods to indicate causality. **(C)** MR analysis forest plot of the association between T2DM and MI. The circles next to each SNP represent causal estimates for each IV, respectively, and the lowest two circles show multiple-instrument MR analysis using Egger regression and inverse-variance weighted methods. Horizontal lines denote 95% CIs. **(D)** MR leave-one-out sensitivity analysis, used to estimate the causal effect of T2DM on MI, each black point represents an IVW, the red point represents the estimated value using all IVs, and the horizontal line represents the 95% confidence interval.

Subsequently, we further evaluated the effect of T2DM on MI. The results showed (**Table 3**, **Figures 2A, B**) that there was a significant causal relationship between T2DM and MI (MR Egger, OR: 0.549, 95%CI: 0.317-0.952, *P* = 0.037; WM, OR: 0.593, 95% CI: 0.400, *P* = 0.010; IVW, OR: 0.767, 95%CI: 0.600-0.980, *P* = 0.034) (**Figure 2C**). Leave-one-out analysis demonstrated the reliability of the results (**Figure 2C**). Finally, for the causal effect between ED and MI, we also performed MR analysis. Surprisingly, although there was a tendency for ED to cause MI, there was no significant difference (*P* > 0.05) (**Supplementary Figure 2**, **Supplementary Table S3**). In conclusion, our 3-step two-sample MR analysis indicated that T2DM causes ED and MI, whereas ED may not cause MI. We also supplement the MR Analysis of relevant data of other populations, including East Asian, Hispanic or Latin American and Mixed (**Supplementary Figure S3**).

**Table 3:** Significant causal relationship between T2DM and male infertility (MI).

exposure	outcome	method	nsnp	β	se	pval	or	or_lci95	or_uci95	Q	Q_pval	egger_intercept	pval_intercept
T2DM	Male infertility	MR Egger	58	0.600	0.281	0.037	0.549	0.317	0.952	37.358	0.974	0.024	0.189
T2DM	Male infertility	WM	58	0.523	0.202	0.010	0.593	0.399	0.880				
T2DM	Male infertility	IVW	58	0.265	0.125	0.034	0.767	0.600	0.980	39.131	0.966		

### rs6585827-suppressed PLEKHA1 expression levels are associated with a lower risk of T2DM

**Figure 3:**
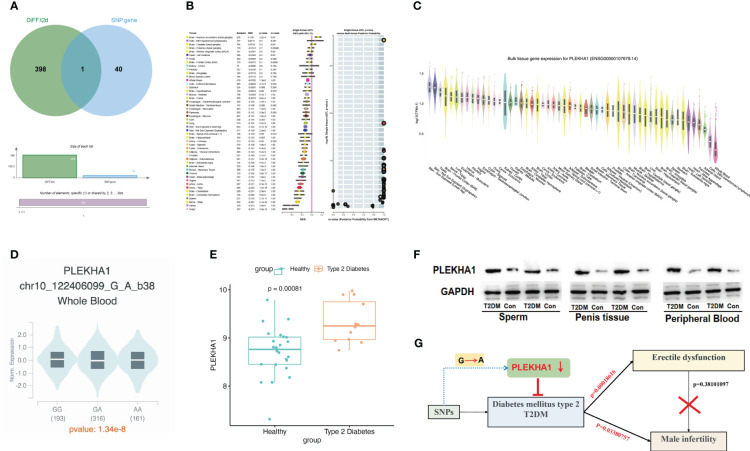
eQTL analysis revealed that rs6585827-suppressed PLEKHA1 expression levels were associated with a lower risk of T2DM. **(A)** Venn diagram; **(B)** PLEKHA1 gene acts as a protective factor in bulk tissue; **(C)** PLEKHA1 gene expression in different tissues; **(D)** PLEKHA1 down-regulated in GA and AA genotypes; **(E)** Expression levels of PLEKHA1 in healthy and T2DM groups. **(F)** The expression of PLEKHA1 protein in the T2DM group of rat semen, penis tissue, and peripheral blood samples. **(G)** Research schematic diagram of this project.

We searched the genes corresponding to 58 SNPs through the GTEx database and found that rs6585827 corresponding to the PLEKHA1 gene is an eQTL variation that affects the expression of this gene. This finding suggested an association between the G to A mutation of rs6585827 and the expression level of the PLEKHA1 gene (**Table 1**). In addition, we also used the gene expression data of the patients in the GSE9006 dataset to intersect the obtained T2DM-related differential genes and the genes corresponding to the SNPs, and only found the PLEKHA1 gene (**Figure 3A**). Clinical and epidemiological findings point to an association between T2DM and osteoporosis. PLEKHA1 was found to be differentially expressed in circulating monocytes of osteoporotic subjects and in PBMCs of diabetic and non-diabetic subjects. Co-genetic assay available for osteoporosis and T2DM (21).

Genome-wide eQTL Study describes the NES (Normalized Effect Size) and 95% CI (Confidence Interval) of eQTL in a single tissue (**Figure 3B**). According to the expression of PLEKHA1 in different tissues in **Figure 3C**, the expression in whole blood was abnormally down-regulated. Subsequently, we compared the expression of PLEKHA1 in the G-to-A chromosome 10 locus 122406099. The results showed that PLEKHA1 was significantly down-regulated in patients with GA genotype and AA genotype compared with 193 patients carrying GG genotype, with a *P* value of 1.34e-8 between the three groups (**Figure 3D**). In addition, combined with **Table 1**, the β value between rs6585827 and PLEKHA1 was -0.036. It can be concluded that the mutation from G to A at rs6585827 leads to the downregulation of PLEKHA1 gene, and the suppressed PLEKHA1 expression level is associated with a lower risk of T2DM. Finally, combined the transcript level data of whole blood samples from 36 T2DM patients in the USA population, the differential analysis of the expression of PLEKHA1 gene between the healthy group and the T2DM group was carried out. The results showed that PLEKHA1 was abnormally upregulated in the T2DM group (*P* < 0.05) (**Figure 3E**). The WB results further validated our analysis, the protein expression of PLEKHA1 was up-regulated in the T2DM group of rat semen, penis tissue, and peripheral blood samples (**Figure 3F**). This result suggests that the PLEKHA1 gene may not be mutated in T2DM patients, but it is associated with the risk of T2DM. **Figure 3G** shows us the main findings of this study.

## Discussions

Unlike risk factors for MI such as lifestyle and environmental factors, we aimed to reveal the association between the endocrine disease T2DM and MI and ED. Through MR analysis, we found that in the European population, T2DM can cause ED and MI. Our findings on the relationship between T2DM and ED are consistent with previous studies. A meta-analysis including 863 men with diabetes and 5385 healthy controls showed a higher prevalence of ED in diabetic patients (22). In addition, a prospective cohort study of 615 Egyptian men also concluded the negative impact of T2DM on ED (23). Bovijn and colleagues similarly found T2DM to be a causal risk factor for ED following univariate MR (18). The findings of Skeldon et al. highlight the importance of ED as a marker of undiagnosed diabetes and should be a trigger to initiate diabetes screening, especially in middle-aged men (24). T2DM is the mechanism by which ED is affected and the pathophysiology is multifactorial, including vascular, neurological and hormonal influences (25). Glycosylation-induced microvascular damage and insufficient oxygen and blood supply to nerves have traditionally been considered etiological (26). Hypogonadism and decreased levels of free and total testes due to T2DM may also be associated with the prevalence of ED (27).

ED and MI are two distinct men’s health concerns, yet there are common risk factors between the two. For example, diabetes may cause damage to blood vessels and nerves, thereby affecting both ED and sperm quality and quantity, resulting in MI (28). Researchers surveyed more than 500 male partners of infertile couples and found that about 1.2% of infertile men suffer from T2DM (29). A new study showed that the prevalence of infertility in men with T2DM has reached 35.1%, which is significantly higher compared with normal participants (30). In addition, this study also found an interesting phenomenon, we did not observe that ED can directly cause MI. This means that MI may be caused by T2DM itself and its related biological mechanisms, rather than the result of ED alone. Even in patients with ED and normal sperm quality and quantity, it is still possible to improve fertility through assisted reproductive technology. In summary, ED may not be the direct cause of MI, but one of the factors affecting sexual behavior. Therefore, in the evaluation and treatment of infertility problems, factors such as erectile function and sperm quality need to be considered comprehensively to find possible causes and formulate corresponding solutions.

The pathogenesis of T2DM is complex, and genetic factors increase the susceptibility to T2D (31, 32). SNP (Single Nucleotide Polymorphism) is one of the most common forms of genetic variation in the genome and the smallest type of variation in DNA sequences. A SNP occurs when a single nucleotide (A, T, C, G) is substituted or inserted/deleted in a DNA sequence. We conducted an eQTL study on the 58 SNPs involved in the MR analysis, and found that rs6585827 corresponding to the PLEKHA1 gene is an eQTL variation that affects the expression of the gene, and the rs6585827 mutation from G to A suppresses the expression level of PLEKHA1 and lower risk of T2DM relevant. Western blot analysis experiments further validated our analysis. PLEKHA1 (also known as TAPP1) encodes a pleckstrin homology domain-containing adapter protein, which is localized to the plasma membrane where it specifically binds phosphatidylinositol 3,4-bisphosphate. At present, the research on this gene mainly focuses on the reports related to age-related macular degeneration, and there are relatively few studies on PLEKHA1 gene in T2DM. The latest study found that PLEKHA1 may represent an important biomarker that may initiate diabetic nephropathy by activating related immune cells (33). Some scholars found that PLEKHA1 mRNA was upregulated in PBMCs of T2DM subjects compared with healthy subjects, and highlighted PLEKHA1 as an important potential pleiotropic gene (21). This is consistent with our analysis that the PLEKHA1 gene is upregulated in T2DM patients and is associated with the risk of T2DM. In the genome, there are two main types of DNA regions: coding regions and noncoding regions. Coding regions contain the genetic sequences required to encode proteins, while non-coding regions contain other types of functional sequences. SNPs in non-coding regions refer to SNPs that occur in non-coding regions of the genome, and they may affect gene regulatory elements, transcription factor binding sites, or other regulatory sequences, thereby affecting intergenic expression and function (34). However, these non-coding SNPs do not directly lead to protein-coding variation. Therefore, genes are not always expressed concordantly in non-coding and coding regions (32). And T2DM, like all complex diseases, is also a disease involving multiple genes and multiple factors, and each gene has a small but cumulative effect (35). Therefore, the expression of PLEKHA1 in patients is often regulated by many factors. This also potentially explains why the rs6585827 mutation in the non-coding region can inhibit the expression of PLEKHA1, while the expression of PLEKHA1 is upregulated in patients. In addition, some studies have reported that PLEKHA1 is relatively highly expressed in the testis [35]. It is worthy of researchers to further explore the intrinsic molecular mechanism of PLEKHA1 abnormality promoting T2DM and its complications.

### Limitation

This study only investigated the impact of SNP on gene expression regulation starting from eQTL analysis. However, different data types reflect various aspects of the same biological process. Therefore, integration of data from different modes such as pQTL (protein quantitative trait loci), sQTL (splice quantitative trait loci), and meQTL (DNA methylation quantitative trait loci) is necessary to assess the genetic influence of SNPs on protein expression, RNA splicing, DNA methylation, and other molecular phenotypes. Comprehensive analysis can provide more information annotation about SNPs, facilitating the interpretation of GWAS results.

In conclusion, this study demonstrates that T2DM has a direct causal effect on ED and MI and anchors the PLEKHA1 gene that is repressed in T2DM due to the rs6585827 mutation. Next, it can be considered to combine clinical sample analysis to further study the association between the expression changes of this gene in T2DM patients and disease progression, and to conduct experimental research on the function and mechanism of PLEKHA1 in the development of T2DM. This will help to identify PLEKHA1 or its related pathways as potential therapeutic targets, so as to develop new therapeutic strategies and reduce the health burden of T2DM and its related complications such as ED and MI.

## Data availability statement

The original contributions presented in the study are included in the article/**Supplementary Material**. Further inquiries can be directed to the corresponding authors.

## Ethics statement

The animal studies were approved by the Ethics Committee (Institutional Review Board) of Shanghai Ruijin Hospital. The studies were conducted in accordance with the local legislation and institutional requirements.

## Author contributions

X-BZ: Data curation, Writing – original draft. Z-HN: Data curation, Writing – review & editing. W-MF: Data curation, Writing – review & editing. C-SS: Writing – review & editing, Supervision. QC: Supervision, Writing – review & editing.

## Supplementary material

Supplementary Table 1 Sample information of GWAS data used in our study.

Supplementary Table 2 GSE9006 characteristic data.

Supplementary Table 3 Causal effect between erectile dysfunction (ED) and male infertility (MI).

Supplementary Figure 1 Characteristics related to T2DM rats. **(A)** Changes of blood glucose, body weight in between Sham and T2DM rats. **(B)** Histomorphological changes of liver, colon, and pancreas tissues.

Supplementary Figure 2 Effect of erectile dysfunction on male infertility.

Supplementary Figure 3 MR Analysis of relevant data of other populations. **(A)** T2DM on erectile dysfunction (ED); **(B)** T2DM on male infertility (MI).
