# Supplementary material for: Type 2 diabetes mellitus and the risk of male infertility: a Mendelian randomization study
Source: Front Endocrinol (Lausanne). 2023 Dec 12;14:1279058. doi: 10.3389/fendo.2023.1279058 (PMC10752377; doi:10.3389/fendo.2023.1279058)
Supplement: Supplementary Table 3 — Causal effect between erectile dysfunction (ED) and male infertility (MI). [file Table_3.docx]

**Table S3 Causal effect between ED and male infertility.**

| **exposure** | **outcome** | **method** | **nsnp** | **β** | **se** | **pval** | **or** | **or_lci95** | **or_uci95** | **Q** | **Q_pval** | **egger_intercept** | **pval_intercept** |
| --- | --- | --- | --- | --- | --- | --- | --- | --- | --- | --- | --- | --- | --- |
| ED | Male infertility | MR Egger | 105 | 0.071 | 0.120 | 0.555 | 1.074 | 0.849 | 1.358 | 96.097 | 0.672 | 0.004 | 0.833 |
| ED | Male infertility | WM | 105 | 0.019 | 0.085 | 0.821 | 1.019 | 0.864 | 1.203 |  |  |  |  |
| ED | Male infertility | IVW | 105 | 0.049 | 0.055 | 0.381 | 1.050 | 0.942 | 1.170 | 96.141 | 0.696 |  |  |
