# Supplementary figures and images for: Type 2 diabetes mellitus and the risk of male infertility: a Mendelian randomization study
Source: Front Endocrinol (Lausanne). 2023 Dec 12;14:1279058. doi: 10.3389/fendo.2023.1279058 (PMC10752377; doi:10.3389/fendo.2023.1279058)

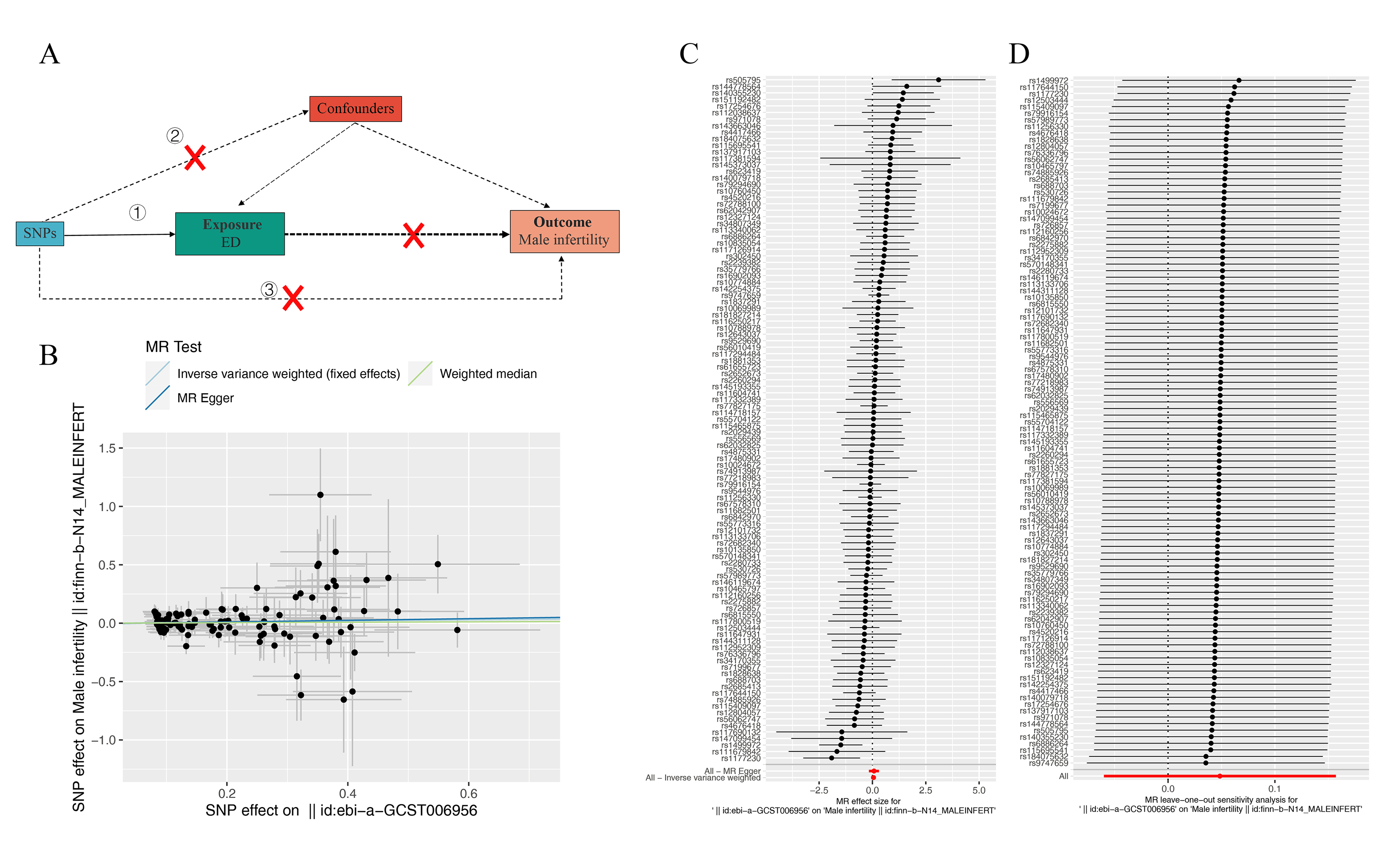

Supplement: Supplementary Figure 2 — Effect of erectile dysfunction on male infertility. [file Image_2.tif]

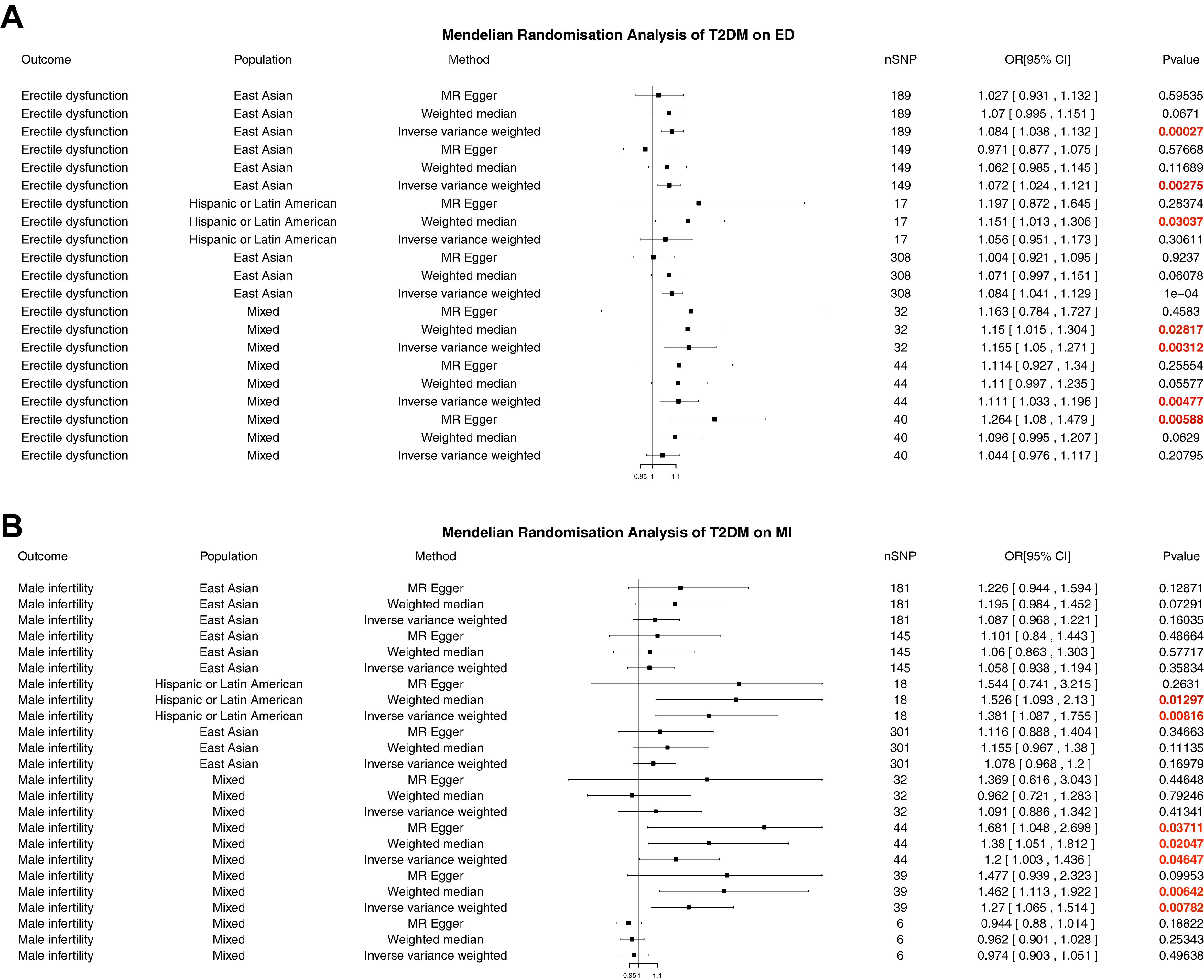

Supplement: Supplementary Figure 3 — MR Analysis of relevant data of other populations. (A) T2DM on erectile dysfunction (ED); (B) T2DM on male infertility (MI). [file Image_3.tif]
